# Analysis of pronuclear zygote configurations in 459 clinical pregnancies obtained with assisted reproductive technique procedures

**DOI:** 10.1186/1477-7827-8-77

**Published:** 2010-06-25

**Authors:** Alessia Nicoli, Francesco Capodanno, Lucia Moscato, Ilaria Rondini, Maria T Villani, Antonella Tuzio, Giovanni B La Sala

**Affiliations:** 1 Sterility Centre "P. Bertocchi", Department of Obstetrics and Gynecology, Arcispedale Santa Maria Nuova, Viale Risorgimento 80, 42100 Reggio Emilia, Italy

## Abstract

**Background:**

Embryos selection is crucial to maintain high performance in terms of pregnancy rate, reducing the risk of multiple pregnancy during IVF. Pronuclear and nucleolar characteristics have been proposed as an indicator of embryo development and chromosomal complement in humans, providing information about embryo viability.

**Methods:**

To correlate the zygote-score with the maternal age and the outcome of pregnancy, we analyzed the pronuclear and nucleolar morphology, the polar body alignment and the zygote configuration in 459 clinical pregnancies obtained by IVF and ICSI in our public clinic in Reggio Emilia, Italy. We derived odds ratios (OR) and Corenfield's 95% confidence intervals (CI). Continuous variables were compared with Student's t-test; P lower than .05 was considered statistically significant.

**Results:**

We observed a significant increase of "A" pronuclear morphology configuration in 38-41 years old patients in comparison to that lower than or equal to 32 years old and a significant decrease of "B" configuration in 38-41 years old patients in comparison to that lower than or equal to 32 and in comparison to that of 33-37 years old.

Related to maternal age we found no significant differences in P1 and in P2 configuration. We found no correlation between zygote-score, embryo cleavage and embryo quality.

**Conclusions:**

Our results confirm the limited clinical significance of zygote-score suggesting that it can not be associated with maternal age, embryo cleavage and embryo quality. The evaluation of embryo quality based on morphological parameters is probably more predictive than zygote-score.

## Background

One of the most important problems in *In Vitro *Fertilization (IVF) treatments is the selection of the best embryos for transfer, a crucial point to maintain high performance in terms of pregnancy rate, reducing at the same time the risk of multiple pregnancy. Nowadays, the quality evaluation and the selection of *in vitro *obtained embryos are made on the basis of morphological parameters involving embryo development, uniformity of blastomeres, percentage of fragmentation, cytoplasmic irregularities, rate of cleavage, blastomeres multinucleation and other visible features [1-5]. These assessments are non-invasive for the embryo development, but on the other hand can not provide any information about embryonic chromosomal arrangement, one of the most relevant aspects of human reproduction, both *in vivo *and *in vitro*. In fact, alterations of chromosomic copy number (aneuploidies) are common in human oocytes and embryos and seem to be mostly implicated in the first-trimester abortions, a complication affecting 50%-70% of all spontaneous conceptions [6]. As described, autosomal trisomies and sex chromosome monosomies, followed by polyploidy and structural rearrangements show a global range from 50% to 80% in first-trimester miscarriages [7,8].

An important contribution in the evaluation of embryo quality seems to come from the pronuclear and nucleolar characteristics, proposed as an indicator of embryo development and chromosomal complement in human fertilized oocytes [9-15]. Nucleoli are the sites of the synthesis of pre-RNA, and ribosomal RNA (rRNA) is necessary for the translational process whereby the embryonic genome becomes fully activated [16]. Despite the high number of studies conducted, to date there are conflicting data about the clinical efficacy of zygote-score: in fact, recent data show that scoring system based on pronuclear morphology seems to provide a good criterion to select embryos for transfer when combined with embryo morphology evaluation on Days 2 and 3 [14] other authors concluded that late parameters (such as the cell number and embryo grade) have a better prognostic value than zygote score when selecting embryos for transfer [17-19].

Finally, to overcome definitely the problem of aneuploidy during IVF, preimplantation genetic diagnosis (PGD) and preimplantation genetic screening (PGS) have been proposed as alternative approaches for embryo selection based on chromosomal arrangement [20], but their efficacy is still debatable [21]. In fact, although several studies have reported an increase in implantation rates and take home baby rates following PGD [22-25] other have failed to show any positive effect of this technique [26-30]. Moreover, one report showed that PGS can have a detrimental effect on pregnancy outcomes [31].

Since several studies seem to confirm that aneuploidy in the human oocytes and embryos tend to increase with the advancing maternal age exceeding 50% by the age 40 years old [32,33], the aim of this study was correlate the pronuclear and nucleolar characteristics with the maternal age and the outcome of pregnancy.

## Methods

### Patients

We analyzed the pronuclear morphology, the nucleolar morphology, the polar body alignment and the zygote configuration in 459 clinical pregnancies obtained by conventional IVF (IVF) (202 clinical pregnancies) and IntraCytoplasmic Sperm Injection (ICSI) (257 clinical pregnancies) between January 2006 and June 2009 in our public clinic in Reggio Emilia, Italy. We stratified the patients according with maternal age in three groups: (i) patients ≤32 years old, (ii) patients 33-37 years old and (iii) patients 38-41 years old.

All of the women and men included in the study had a normal karyotype, normal hormonal assessments, negative vaginal or urethral cultures, and had no malignancy or systemic diseases. The maternal age was 35.9 ± 4.0. Before ovarian stimulation, every woman underwent clinical and psychological examinations as well as transvaginal ultrasound and hormonal evaluations and all male patients underwent a preliminary sperm analysis.

The analyzed IVF and ICSI cycles were performed under the reproductive Italian law 40/2004 that allowed the insemination of not more than three oocytes per cycle with the subsequent transfer of all the obtained embryos [34].

The study was approved by the local ethics committee.

### Ovarian stimulation, oocyte retrieval and sperm analysis

Ovarian down regulation was obtained with a long luteal leuprolide acetate protocol (Enantone 3.75 mg; Takeda, Milano, Italy) or leuprolide acetate micro dose flare protocol (Enantone 0.1 mg; Takeda) [35].

Fourteen to 20 days were needed for complete ovarian suppression, as assessed by serum estradiol concentrations (E_2 _<50 pg/mL) and ovarian ultrasound (no follicles >10 mm). When the suppression criteria were satisfied, recombinant follicle-stimulating hormone (75 IU FSH; Gonal F; Serono, Rome, Italy) was started, using three to six ampule (225-450 IU/day) for the following 5 days. The ovarian response was then monitored daily by transvaginal ultrasound and serum E_2 _assays. The recombinant FSH dosage was adjusted individually according to the ovarian response as judged by both serum E_2 _levels and follicular growth. When one or more follicles >17 mm in diameter and serum E_2 _levels of 200 pg/mL per follicle <15 mm in diameter were obtained, 10,000 IU human chorionic gonadotropin (hCG) was administrated intramuscularly. Thirty-six hours after hCG administration, we performed oocyte retrieval by ultrasound-guided transvaginal aspiration.

All patients undergoing embryo transfer received supplemental progesterone intramuscularly (100 mg/day for 15 days; Prontogest, AMSA, Milano, Italy), or vaginally (400 mg/day for 15 days; Prometrium; Rottapharm, Milano, Italy).

### Semen preparation, oocyte insemination and embryo culture

Semen samples were collected by masturbation after 3 to 5 days of abstinence. The preparation for IVF or ICSI was performed as described elsewhere [36]. Briefly, an appropriate aliquot of fresh semen was diluted with 10 mL of buffer medium (Cook IVF, Melbourne, Australia); after centrifugation (10 minutes at 800 × g at room temperature), the supernatant was removed and replaced by another 5 mL of buffer medium. After a second centrifugation, the supernatant was once again removed, and the pellet was overlaid with 1 mL of medium and incubated (37°C, 6% CO_2 _in air) to separate by swim up.

After liquefaction, in case of poor semen (sperm concentration <1 × 10^6^/mL), the sample was concentrated by one centrifugation (1500 × g) and the pellet removed in 1 mL of medium. All conventional IVF procedures had a percentage of normal form sperms ≥5% (according to strict Kruger's criteria) and/or a number of activated spermatozoa ≥1.5 × 10^6^/ml after capacitation, and oocytes were cultured individually and inseminated in microdrops of fresh medium (Cook, IVF, Melbourne, Australia), under mineral oil. For ICSI, following removal of the oocyte's surrounding cumulus and corona cells, nuclear maturation assessment was performed using an inverted microscope to ensure the injection of metaphase II oocytes exclusively. The ICSI procedure was performed as reported by Palermo and coworkers [37].

### Assessment of fertilization, embryo cleavage and embryo quality

Oocyte fertilization was assessed 18 to 20 hours after IVF or ICSI by confirmation of the presence and location of two pronuclei (2PN) and the alignment of nucleolar precursor bodies (NPB), concomitantly with assignment of the pronuclear morphology score [9]. The observation of 2PN was performer using an inverted microscope with Hoffman modulation contrast using a magnification of ×400 (TE 2000 U, Nikon Corp., Tokyo, Japan).

**Figure 1:**
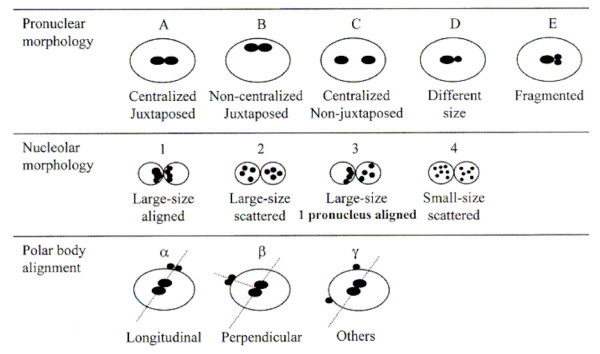
**Different configurations used for pronuclear morphology, nucleolar morphology and polar body alignement assessment**. Pronuclear morphology is classified as A-E, nucleolar morphology is classified as 1-4 and polar body alignement is classified as α, β or γ (9).

For pronuclear morphology score five different configurations regarding pronuclear morphology (A-E), four configurations regarding nucleolar morphology (1, 2, 3, 4) and three configurations regarding polar body alignment (α, β, γ) were adopted as reported by Gianaroli and coworkers [9] (Figure 1).

**Figure 2:**
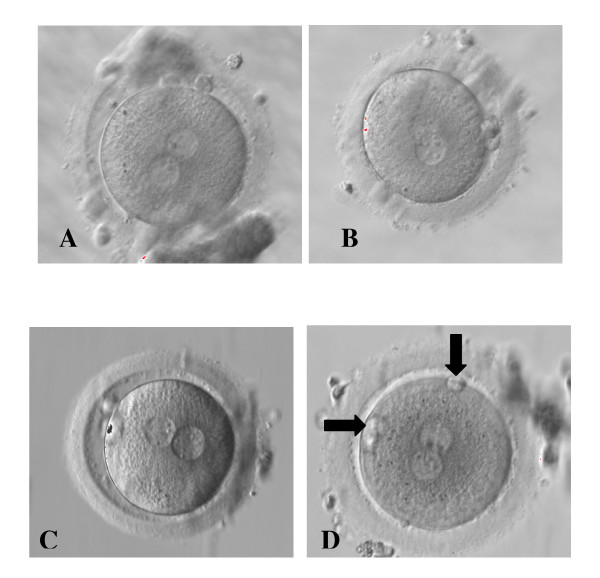
**Zygotes showing different distribution of NPB in the 2PN and different PB aligment (Original magnification ×400)**. Zygotes observed after 18-20 hours after insemination. (A) 2PN centralized and juxtaposed, NPBs aligned on the side of the 2PN, longitudinal PB alignment. (B) 2PN centralized and juxtaposed, NPBs dispersed in the 2PN, perpendicular PB alignment. (C) 2PN centralized and juxtaposed, NPBs non polarized with dispersed or not completely aligned NPBs in the 2PN, longitudinal PB alignment. (D) 2PN centralized and juxtaposed, NPBs aligned on the side of the 2PN, neither longitudinal nor perpendicular PB alignment. Black narrows indicate the PBs. (A) and (B) were examples of zygotes included in Pattern 1 group, while (C) and (D) were examples of zygotes included in Pattern 2 group.

We classified any zygote (Figure 2) as Pattern 1 (P1) or Pattern 2 (P2) on the basis of 2PN zygote score: we considered P1 all zygotes classified as A1α, A2β and A3β, and P2 all the other zygotes (Figure 1). As reported by Gianaroli et al [9], zygote configurations A1α, A2β and A3β correspond with an higher prognostic value for embryo development and pregnancy rate. On the contrary, all other zygote configurations correspond with a lower prognostic value.

Embryo cleavage and embryo quality were evaluated 48 hours after insemination. For embryo quality assessment we followed the method of Plachot et al [38]. For embryo cleavage evaluation we recorded a numeric score for regularity of the blastomeres (dimension, shape), percentage of fragmentation, and appearance of the cytoplasm [39]. A score 0 was given if the blastomeres were regular in shape and dimension, the embryo did not show fragmentation, and the cytoplasm was homogeneous without vacuoles or granulations. Embryos with total score 0 were classified as ''excellent quality embryos,'' whereas in the absence these characteristics, the embryos were given different scores and considered to be ''non excellent'' embryos. All available embryos were trasnferred 48 hours after insemination (Day 2).

### Establishment of clinical pregnancy

Clinical pregnancy was defined as at least one fetus with a positive heartbeat revealed by transvaginal sonography 4 or 5 weeks after embryo transfer. Implantation rate was defined as the number of gestational sacs on ultrasound as a percentage of the embryos transferred.

### Analysis

For all cycles we evaluated maternal age, sperm concentration, sperm motility percentage and sperm morphology. Moreover, total number of recovered and injected oocytes, cleavage rate and total number of embryos obtained were evaluated. For each zygote we evaluated pronuclear morphology, nucleolar morphology, polar body alignment, zygote pattern, embryo cleavage and embryo quality. Then, we evaluated implantation rate.

All data were entered in Excel. We derived odds ratios (OR) and Corenfield's 95% confidence intervals (CI). Continuous variables were compared with Student's t-test; P < .05 was considered statistically significant.

## Results

In the present study we analyzed the pronuclear and nucleolar morphology, the polar body alignment and the zygote configuration in 459 clinical pregnancies obtained by IVF (202 clinical pregnancies) and ICSI (257 clinical pregnancies).

As showed in Table 1, we did not found statistically significant differences in terms of IVF and ICSI distribution in our population, retrieved oocytes per cycle, inseminated oocytes, 2 pronucleate zygotes, and implantation rate between patients ≤32 years old, 33-37 years old and 38-41 years old. On the contrary, we found a statistically significant increase of fertilization rate in patients 33-37 years old in comparison to patients ≤ 32 years old (84.0% vs. 78.4%, O.R. 0.692;C.I. 95% 0.487-0.984) and an increase in cleaved and transferred embryos in patients 33-37 years old in comparison to patients ≤32 years old (p < 0.05).

**Table 1:** Biological and clinical results in IVF cycles.

Parameter	≤32	33-37	38-41	Total	Statistics
**Cycles with clinical pregnancy**	107	196	156	459	
**IVF**	41	90	71	202	n.s.*
**ICSI**	66	106	85	257	n.s.*
**Patients with clinical pregnancy**	105	182	149	436	
**Maternal age (mean ± SD)**	30.3 ± 2.4	35.7 ± 1.4	40.0 ± 1.3	35.9 ± 4.0	
**IVF attemps (mean)**	(1.0)	(1.1)	(1.1)	(1.1)	
**Retrieved oocytes°(mean ± SD)**	728 (6.8 ± 3.5)	1273 (6.5 ± 3.5)	954 (6.1 ± 3.1)	2955 (6.4 ± 3.4)	n.s
**Inseminated oocytes°(mean ± SD)**	306 (2.9 ± 0.6)	569 (2.9 ± 0.6)	451 (2.9 ± 0.8)	1326 (2.9 ± 0.7)	n.s
**2 pronucleate zygotes°(mean ± SD)**	240 (2.2 ± 0.8)	478 (2.4 ± 0.7)	360 (2.3 ± 0.8)	1078 (2.3 ± 0.8)	n.s.
**2 pronucleate zygotes°(%)**	78.4	84.0	79.8	81.3	0.692 (0.487-0.984)§
**Cleaved and transferred embryos°(mean ± SD)**	235 (2.2 ± 0.8)	474 (2.4 ± 0.7)	353 (2.3 ± 0.8)	1062 (2.3 ± 0.8)	<0.05**
**Implantation rate°(%)**	107/235 (45.5)	196/474 (41.3)	156/353 (44.2)	459/1062 (43.2)	n.s.

* not statistically significant both for maternal ages and for insemination technique

°all values referred to cycle number

§ ≤32 vs. 33-37, O.R. (C.I. 95%)

** ≤32 vs. 33-37, p value significative < 0.05

The assessment of pronuclear morphology (Table 2) showed a statistically significant increase in clinical pregnancies in "A" configuration in 38-41 years old patients in comparison to ≤ 32 years old patients (79.4% vs.. 72.0%, O.R. 0.667;C.I. 95% 0.456-0.976) and in comparison to 33-37 years old patients (79.4% vs.. 69.8%, O.R. 0.600;C.I. 95% 0.434-0.828). The same trend was observed in ongoing pregnancies/deliveries (Table 2).

**Table 2:** Pronuclear morphology in zygotes related to the maternal age.

Clinical pregnancies					Ongoing pregnancies/deliveries			
	≤32	33-37	38-41	O.R. (C.I. 95%)	≤32	33-37	38-41	O.R. (C.I. 95%)
**A**	172 (72.0)	330 (69.8)	281 (79.4)	0.667 (0.456-0.976)* 0.600 (0.434-0.828)°	125 (69.1)	275 (67.9)	195 (81.2)	0.511 (0.328-0.808)* 0.484 (0.330-0.711)°
**B**	55 (23.0)	123 (26.0)	53 (15.0)	1.698 (1.118-2.578)* 1.996 (1.398-2.849)°	44 (24.3)	116 (28.3)	34 (14.2)	2.099 (1.278-3.448)* 2.424 (1.592-3.687)°
**C**	9 (3.8)	11 (2.3)	11 (3.1)	n.s.	9 (5.0)	9 (2.2)	8 (3.4)	n.s.
**D**	3 (1.3)	9 (1.9)	9 (2.5)	n.s.	3 (1.6)	6 (1.5)	3 (1.2)	n.s.
**E**	0 (0.0)	0 (0.0)	0 (0.0)	n.s.	0 (0.0)	0 (0.0)	0 (0.0)	n.s.
**All zygotes**	239	473	354		181	406	240	

* ≤32 vs. 38-41

°33-37 vs. 38-41

Moreover, the pronuclear morphology analysis showed an opposite trend in "B" configuration (Table 2): in fact, we observed a statistically significant decrease in clinical pregnancies in "B" configuration in 38-41 years old patients in comparison to ≤ 32 years old patients (15.0% vs. 23.0%, O.R. 1.698;C.I. 95% 1.118-2.578) and in comparison to 33-37 years old patients (15.0% vs. 26.0%, O.R. 1.996;C.I. 95% 2.398-2.849). Similarly, we found the same trend in ongoing pregnancies/deliveries (Table 2).

We did not observe any statistically significant differences in "C", "D" and "E" configurations related to maternal age both in clinical pregnancies and in ongoing pregnancies/deliveries (Table 2).

In nucleolar morphology analysis, we found no statistically significant differences in all considered configurations (1, 2, 3, 4) related to maternal age both in clinical pregnancies and in ongoing pregnancies/deliveries (Table 3).

**Table 3:** Nucleolar morphology related to the maternal age.

Clinical pregnancies					Ongoing pregnancies/deliveries			
	≤32	33-37	38-41	O.R. (C.I. 95%)	≤32	33-37	38-41	O.R. (C.I. 95%)
**1**	134 (56.1)	274 (57.9)	193 (54.5)	n.s.	96 (53.0)	230 (56.6)	132 (55.0)	n.s.
**2**	37 (15.5)	65 (13.7)	60 (16.9)	n.s.	32 (17.7)	62 (15.3)	37 (15.4)	n.s.
**3**	53 (22.2)	106 (22.4)	86 (24.3)	n.s.	41 (22.6)	88 (21.7)	61 (25.4)	n.s.
**4**	15 (6.3)	28 (5.9)	15 (4.2)	n.s.	12 (6.6)	26 (6.4)	10 (4.2)	n.s.
**All zygotes**	239	473	354		181	406	240	

In Table 4 are reported the results of polar body alignment analysis. We found a statistically significant increase of "β" configuration in 33-37 years old patients compared with 38-41 years old patients both in clinical pregnancies (31.1% vs. 37.9%, O.R. 0.740;C.I. 95% 0.554-0.989), and in ongoing pregnancies/deliveries. On the contrary, in "α" and "γ" configurations we found no statistically significant differences related to maternal age in clinical pregnancies and in ongoing pregnancies/deliveries (Table 4).

**Table 4:** Polar body alignment related to the maternal age

Clinical pregnancies					Ongoing pregnancies/deliveries			
	≤32	33-37	38-41	O.R. (C.I. 95%)	≤32	33-37	38-41	O.R. (C.I. 95%)
**α**	118 (49.4)	247 (52.2)	165 (46.6)	n.s	91 (50.3)	213 (52.5)	111 (46.2)	n.s
**β**	82 (34.3)	147 (31.1)	134 (37.9)	0.740 (0.554-0.989)°	62 (34.2)	123 (30.3)	94 (39.2)	0.675 (0.483-0.943)°
**γ**	39 (16.3)	79 (16.7)	55 (15.5)	n.s	28 (15.5)	70 (17.2)	35 (14.6)	n.s.
**All zygotes**	239	473	354		181	406	240	

°33-37 vs. 38-41

**Figure 3:**
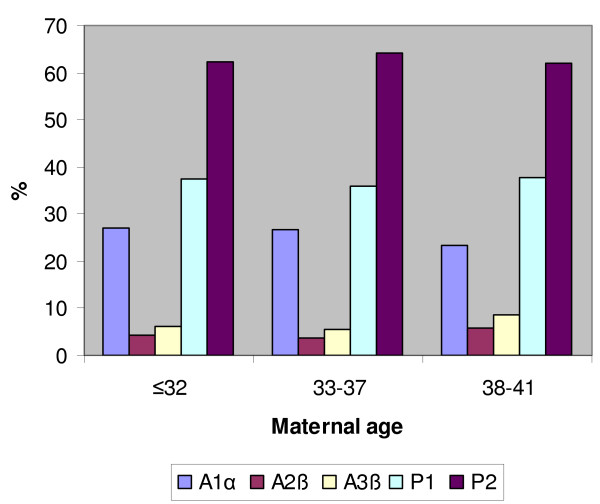
**P1 and P2 distributions in ongoing pregnancies/deliveries group**. Pattern 1 (P1) and Pattern 2 (P2) distributions in ongoing pregnancies/deliveries groups. We classified any zygote as P1 or P2 on the basis of 2PN zygote score: we considered P1 all zygotes classified as A1α, A2β and A3β, and P2 all the other zygotes.

Moreover, we found no statistically significant differences correlating P1 (A1α, A2β and A3β) and P2 (pool of all the other configurations) configurations with embryo cleavage and embryo quality in clinical pregnancies (Table 5). We observed a similar trend in ongoing pregnancies/deliveries (data not showed). Finally, we found no statistically significant differences in P1 and P2 configurations related to maternal age in clinical pregnancies. Similarly, we found the same result in ongoing pregnancies/deliveries (Figure 3).

**Table 5:** Zygote morphology related to embryo cleavage and embryo quality.

	P1		P2		
	**≥4 blastomeres**	**Excellent Quality**	**≥4 blastomeres**	**Excellent Quality**	**OR (IC 95%)**

**<32**	79/89 (88.8)	40/89 (44.9)	123/150 (82.0)	56/150 (37.3)	n.s
**33-37**	134/170 (78.8)	65/170 (38.2)	224/303 (73.9)	113/303 (37.3)	n.s
**38-41**	101/128 (78.9)	55/128 (43.0)	185/226 (81.9)	94/226 (41.6)	n.s
**Total**	314/387 (81.1)	162/387 (41.9)	532/679 (78.4)	258/679 (38.0)	

## Discussion

One of the main items of IVF treatments is maintain an high performance in terms of pregnancy rate reducing at same time the multiple gestations; the selection of the best embryo/embryos to transfer is a key step to achieve this objective. Nowadays, the embryo selection is mainly performed on the basis of visible morphological parameters [2-5], but this assessment can not provide any information about embryonic aneuploidies, a problem increasing with the advancing maternal age [32,33], and probably implicated in the most of the first-trimester spontaneous miscarriages [8]. The evaluation of the pronuclear and nucleolar characteristics (zygote-score) has been proposed as an indicator of embryo development and chromosomal complement in human fertilized oocytes [13-19]. The study of human zygote seem to provide important information about embryonic chromosomal arrangements, even if, to date, there is no definitive scientific evidence about its clinical efficacy [17,18]. Recent data seem to consider pronuclear evaluation as a good criterion when combined with embryo morphology evaluation on Days 2 and 3 [14], other authors conclude that late parameters (number of blastomeres and embryo grade) have a better prognostic value than zygote score [19].

In the present study, we have evaluated the clinical significance of zygote-score related to maternal age in patients submitted to ART cycles successfully, obtaining a clinical pregnancy. We observed that all parameters analyzed (pronuclear morphology, nucleolar morphology, polar body alignment and zygote configuration) were generally uniformly distributed in patients ≤32 years old, 33-37 years old and 38-41 years old, showing only few differences related to maternal ages.

Studying the pronuclear morphology, we observed a statistically significant increase in "A" configuration, with a concomitant decrease in "B" configuration, in 38-41 years old in comparison to ≤37 years old patients, both in clinical pregnancies and in ongoing pregnancies/deliveries groups. Due to our unique population characteristics (all patients with clinical pregnancy and maternal age subdivision), it is difficult to compare our results with others present in literature, nevertheless these results appear to corroborate with what has been reported in literature [9]. In agreement with previously reported data [19], our analysis of nucleolar morphology showed no statistically significant differences in relation to maternal ages, both in clinical pregnancy and in ongoing pregnancy/deliveries groups. This result is in contrast with recently published data showing a correlation between pronuclear morphology and maternal ages [15]. We believe that the discrepancy could be related to the different sample size and to the different maternal age between our patients and those analyzed by Maille and coworkers. Moreover, in contrast with Maille and coworkers, we included in the study population clinical pregnancies obtained both with IVF and ICSI. Again, more recent data seem to confirm the poor clinical significance of nucleolar morphology during embryo selection, reporting no correlations between this parameter and implantation rate [19].

Finally, studying the last zygote parameter - polar body alignment - related to maternal age, our data showed a statistically significant decrease of "β" configuration in patients 38-41 years old, both in clinical pregnancies and in ongoing pregnancies/deliveries group. We can speculate that polar body alignment, usually evaluated during zygote-score assessment, not appear to have a prognostic value in terms of embryo viability and pregnancy rate. Than, it can not be considered a good tool to select the best embryo to transfer.

This assumption seems to be confirmed by the evaluation of the last parameter considered in our analysis: the whole zygote configuration (pronuclear and nucleolar morphologies plus polar body alignment). In particular, the total absence of statistically significant differences between zygote configurations P1 and P2 grouped by maternal ages, probably provide an additional evidence about the limited importance of zygote-score in assisted reproduction outcomes, as proposed by our previously data and confirmed by most recently published results [18,19]. Our results showed any statistical significance in term of embryo cleavage and embryo quality related to P1 or P2 configurations both in clinical pregnancies and in ongoing pregnancies/deliveries.

## Conclusions

In conclusion, the results of our study confirm that zygote-score assessment have a limited clinical significance in the choice of the best embryos to transfer during IVF treatments. Probably, as proposed by other authors [19,40] and suggested in this study, the evaluation of embryo quality performed on the basis of number of blastomeres, embryo morphological characteristics and grade is more predictive than zygote-score.

## Competing interests

The authors decleare that they have no competing interests.

## Authors' contributions

AN conceived the study and the design. FC carried out the bibliographic research and drafted the manuscript. LM carried out statistical analysis. IR analyzed the results. MTV and AT helped to draft the manuscript. GBLS participated in the study as project supervisor coordinator with its critical revision of the manuscript. All authors read and approved the final manuscript.
